# Trend of electroconvulsive therapy use and its relationships with clinical characteristics from a large psychiatric center in China

**DOI:** 10.3389/fpsyt.2025.1508044

**Published:** 2025-06-03

**Authors:** Wei Li, Na Hu, Xiaoxiao Gao, Yanying Song, Rongzhen Zhang, Shiyou Sun, Jinghui Tong, Yang Shen, Yongjun Yu, Kebing Yang, Yan Chen, Jiaqi Song

**Affiliations:** ^1^ Beijing Huilongguan Hospital, Beijing, China; ^2^ Huilongguan Clinical Medical School, Peking University, Beijing Huilongguan Hospital, Beijing, China

**Keywords:** electroconvulsive therapy, inpatients, China, daily living abilities, clinical characteristic

## Abstract

**Background:**

Recent studies on electroconvulsive therapy (ECT) have reported inconsistent frequencies of ECT use in various countries. Therefore, this study aimed to investigate the trends of ECT use in a large psychiatric center in China over 6 consecutive years.

**Methods:**

A total of 22,120 inpatients, aged 18–59 years, admitted during the period 2015–2020 to a large grade-A tertiary psychiatric center in Beijing were enrolled in this retrospective study. Demographic and clinical data including vital signs; daily living abilities(ADL); emergency referrals; psychiatric and physical prescriptions were collected from an electronic medical records system.

**Results:**

In all, 2,213 (10.0%) inpatients received ECT, with an average number of sessions of 10.3 ± 6.6. There were no significant differences between the ECT and non-ECT groups in terms of educational level, marital status, length of hospital stay, and blood pressure. After using the propensity score matching (PSM) method, Multiple logistic regression analysis revealed that ECT use was independently associated with married/cohabitating (OR = 1.21, 95% CI: 1.03-1.43); few hospitalizations (OR = 0.96, 95% CI: 0.93-0.99); unemployed (OR = 1.43, 95% CI: 1.16-1.76); emergency referral (OR = 1.62, 95% CI: 1.36-1.93); increased use of antipsychotics (OR = 2.63, 95% CI: 1.88-3.68), mood stabilizers (OR = 1.30, 95% CI: 1.01-1.67), antidepressants (OR = 1.40, 95% CI: 1.13-1.73), and trihexyphenidyl (OR = 1.30, 95% CI: 1.05-1.50); reduced use of hypoglycemic drugs (OR = 0.64, 95% CI: 0.45-0.83); fast heart rate (OR = 1.01, 95% CI: 1.01-1.02); and severe impairments in ADL. Compared with that in 2015 (13.2%), ECT use decreased annually from 2016 (12.4%) to 2019 (9.6%), especially in 2020 (5.7%), given the impact of the COVID-19 pandemic in China.

**Conclusions:**

The ECT usage and year-by-year decrease in ECT use in this study were consistent with the recent trends in other regions. Patients with the married/cohabitating, unemployed, and emergency-referral, unstable vital signs, more severe disability received ECT for quick alleviation of their conditions.

## Introduction

Electroconvulsive therapy (ECT) is considered a highly effective nonpharmacological intervention for patients with severe psychiatric disorders (1). With the rapid development of psychiatry, there is a growing body of evidence indicating that comprehensive treatment patterns, including medication, psychotherapy, physical therapy, and social rehabilitation therapy, are currently the main clinical strategies. ECT plays a crucial role in the acute phase of several serious mental illnesses (2), and its role is incomparable to the roles of medication and psychological therapy (3).

ECT has been used clinically for nearly 90 years since it was first demonstrated in Rome in 1938. The basic mechanism of ECT is the application of a brief electric current to the patient’s scalp to induce a generalized seizure that, in turn, alleviates severe psychiatric symptoms (4). As the findings of Leiknes and colleagues’ 2012 review (5), Regenold and colleagues’ 2022 survey (6), the ECT performed today has remained roughly the same as that in the past decade since the early 20th century. ECT is considered a strict therapeutic strategy for patients with a high risk of suicide, impulsiveness, aggressive tendencies, and resistance to pharmacotherapy (7, 8).

Information on the patterns of ECT use across countries has been inconsistent (5, 9). There had been sustained declining use of ECT in the United States from 1993 to 2009 (10). In contrast, a large survey found that 30% of the patients with mood disorders in southern China received ECT – while the rate is lower than that reported in Beijing (33.6% in 2007 and 61.8% in 2013), it is far higher than the rates reported in European countries and the United States (11, 12). However, with the popularization of ECT usage in mental disease and stigmatization of ECT, it is necessary to update the information on the use of ECT in China in the recent 5 years or even longer (5).

This study retrospectively investigated the ECT use and the correlated demographic and clinical factors by examining data from a large psychiatric hospital in China for a period of 1 January 2015 to 31 December 2020.

## Methods

### Subjects and ECT setting

The study was conducted at Beijing Huilongguan Hospital, Huilongguan Clinical Medical School, Peking University, China. This center has 1369 beds and is one of the largest grade-A tertiary psychiatric hospitals in Beijing and even in China. In this hospital, ECT is primarily administered to inpatients. For adult patients, the ECT course usually comprises 6–12 sessions under general anesthesia, over 2–3 weeks (13). The anesthesia and muscle relaxation is induced with propofol (1–1.5 mg/kg) accompanied by succinylcholine (0.3–0.7 mg/kg) and oxygenation. The intensity of ECT is based on two-thirds of the patient’s age (14).

ECT is delivered via the electrodes of a MECTA spectrum M5000Q stimulator (MECTA Corp, Tualatin, Oregon, USA) that are placed on the bilateral temporal lobes. The following ECT parameters are used: maximum charge delivered, 504 mC; output current, 0.9 A; frequency, 10 to 70 Hz; pulse width, 1.0 ms; maximum stimulus duration, 8 s (15). After each ECT session, the patient is transferred to the recovery room for close monitoring.

### Collection of demographic and clinical characteristics

The information of all patients aged 18–59 years who were receiving adult psychiatric services was retrieved from the electronic medical record system (EMRS). The following inpatient data were collected: basic demographic and clinical characteristics including the diagnoses, psychiatric and physical (non-psychiatric) prescriptions, emergency referrals, vital signs, and ADL performance.

Psychiatric diagnoses were divided into four categories: schizophrenia or other psychotic disorders (SZ), bipolar disorders (BD), major depressive disorders (MDD), and others. The diagnosis was confirmed by two experienced psychiatrists according to the diagnostic criteria of the International Statistical Classification of Diseases and Related Health Problems (ICD-10). If a patient had more than one diagnosis, only the primary diagnosis was used.

Vital signs, including systolic blood pressure (SBP), diastolic blood pressure (DBP), and heart rate, was considered one of the crucial indicators for indirectly assessing the stability of physical conditions. Unstable physical conditions can also have an negative impact on mental illnesses, such as the blood pressure variability increasing the risk of anxiety (16). These indicators were recorded and calculated as the average of measurements before each ECT using a unified electronic sphygmomanometer. The accuracy of an electronic sphygmomanometer is usually verified every 6 months. Generally, the blood pressure and heart rate in the right upper limb is measured while the patient is seated.

ADL performance, an important outcome evaluated in this study, was assessed using the Barthel ADL Index at admission and discharge (17). The ADL index includes 10 items (feeding, transfer, personal hygiene, toilet use, bathing, walking, going up and down stairs, dressing, stool, and urination). ADL ability was classified as follows according to the ADL score (range: 0–100): ADL self-care (100), mild ADL disability (65–95), moderate ADL disability (45–60) and severe ADL disability (0–40). Low scores indicate impairments in ADL (18). Cronbach’s alpha for ADL was 0.94 (19).

The authors (X.X.G and J.H.T.) and a technician responsible for EMRS collected the data and established the study database. The study design was approved by the Ethics Committee of Beijing Huilongguan Hospital.

### Statistical analysis

All data were analyzed using SPSS software (version 22.0; SPSS Inc., Chicago, IL, USA). Basic demographic and clinical characteristics between the ECT and non-ECT groups were compared using the chi-square test for categorical variables and the Mann–Whitney U test for continuous non-normally distributed variables, as appropriate. To reduce bias and confounding variables, propensity score matching (PSM) analysis was conducted on participants based on gender and age, with matching pairs on 1:1. SPSS software was used to calculate conditional logistic regression between the paired data. The odds ratio (OR) and 95% confidence interval (CI) for each variable were calculated. Finally, the trends and use rate in ECT among the four disease types from 2015 to 2020 were analyzed separately. The level of significance was set at 0.05 (two-tailed).

## Results

The data of 22,120 patients who were hospitalized during the study period and met the study criteria were analyzed. The mean age was 41.8 ± 12.7 years, the length of current hospital stay was 71.8 ± 68.2 days, and the number of hospitalizations was 7.4 ± 7.9; 57.1% of the sample consisted of men; 59.5%, 14.7%, 12.6%, and 13.2% had a primary diagnosis of SZ, BD, MDD, and other, respectively. The most frequently prescribed medication class was antipsychotics (89.6%), followed by trihexyphenidyl (30.7%), antidepressants (25.8%), mood stabilizers (21.9%), and cognitive-enhancing drug (0.4%). Commonly prescribed drugs for physical diseases included lipid-lowering drugs (24.0%), hypoglycemic drugs (11.6%), and anti-hypertensive drugs (11.1%).

**Table 1** outlines the demographic and clinical characteristics of the study sample and the results of the comparative analyses between the ECT and non-ECT groups. Of these, 2213 individuals (10.0%) underwent ECT. The corresponding proportions were 17.8% for BD, 17.8% for MDD, 8.2% for SZ, and 2.1% for other diagnoses (P<0.001). There were significant differences in all demographic and clinical characteristics, except education, marital status, nationality, religion, current hospital stay, and DBP and SBP before ECT between the two groups (**Table 1**).

**Table 1:** Demographic and characteristics of the study sample.

Characteristics	The whole sample(n=22120)		Non-ECT group (n=19907)		ECT group (n=2213)		Statistics		
	N	%	N	%	N	%	*X^2^*	Df	*P*
Male, N(%)	12629	57.1	11818	59.4	811	36.6	419.6	1	<0.001
Education(Undergraduate)	11510	52.0	10411	52.3	1099	49.7	3.5	1	0.61
Married/cohabitating	11163	50.5	10053	50.5	1110	50.2	0.04	1	0.85
Han nationality	21597	97.6	19432	97.6	2165	97.8	0.4	1	0.52
Religion(No religious belief)	20892	94.4	18812	94.5	2080	94.0	0.5	1	0.45
Local resident	13985	63.2	13039	65.5	946	42.7	443.4	1	<0.001
Employed	18556	83.9	16791	84.3	1765	79.8	31.1	1	<0.001
Emergency in hospital	4240	19.2	3438	17.3	802	36.2	462.6	1	<0.001
Primary psychiatric diagnosis							654.5	3	<0.001
Schizophrenia-spectrum disorders	13155	59.5	12077	60.7	1078	48.7			
Bipolar disorder	3259	14.7	2680	13.5	579	26.2			
Major depression	2779	12.6	2285	11.5	494	22.3			
Others	2927	13.2	2865	14.4	62	2.8			
Psychiatric drugs									
Use of antipsychotics	19818	89.6	17758	89.2	2060	93.1	32.2	1	<0.001
Use of mood stabilizers	4844	21.9	4152	20.9	692	31.3	126.3	1	<0.001
Use of antidepressants	5710	25.8	4850	24.4	860	38.9	218.6	1	<0.001
Use of cognitive-enhancing drug	88	0.4	86	0.4	2	0.1	5.9	1	0.019
Use of trihexyphenidyl	6796	30.7	5938	29.8	858	38.8	74.8	1	<0.001
Physical disease drugs									
Use of antihypertensive drugs	2248	11.1	2289	11.5	159	7.2	37.7	1	<0.001
Use of lipid-lowering drugs	5306	24.0	4881	24.5	425	19.2	30.9	1	<0.001
Use of hypoglycemic drugs	2576	11.6	2482	12.5	94	4.2	130.8	1	<0.001
Year							127.8	5	<0.001
2015	3376	15.3	2931	14.7	445	20.1			
2016	3570	16.1	3128	15.7	442	20.0			
2017	3926	17.7	3537	17.8	389	17.6			
2018	4163	18.8	3788	19.0	375	16.9			
2019	4106	18.6	3713	18.7	393	17.8			
2020	2979	13.5	2810	14.1	169	7.6			
	M	SD	M	SD	M	SD	*Z*		*P*
Age	41.8	12.7	42.5	12.6	35.7	12.2	-23.8		<0.001
Current hospital stay (days)	71.8	68.2	72.4	69.1	66.5	59.7	-0.8		0.45
No. of hospitalizations	7.4	9.7	7.9	9.9	2.6	3.3	-30.3		<0.001
Heart rate (/min)	81.2	12.4	81.0	12.5	83.0	12.1	-9.5		<0.001
SBP before ECT course (mmHg)	117.7	12.9	117.8	12.8	117.4	13.9	-0.8		0.41
DBP before ECT course (mmHg)	76.0	8.6	76.0	8.5	76.0	9.4	-0.2		0.89
ADL total at admission	91.6	10.6	92.2	10.1	87.2	13.1	-20.1		<0.001
ADL total at discharge	96.1	8.2	96.0	8.2	96.9	7.6	-8.5		<0.001
Changes in ADL total	4.2	8.4	3.8	7.5	9.7	12.3	-30.4		<0.001
No. Sessions of ECT					10.3	6.6			
The intensity of ECT (J)					27.7	15.2			
Seizure duration (s)					49.2	23.1			

SBP, systolic blood pressure; DBP, diastolic blood pressure; ADL, daily living abilities.

After the PSM based on gender and age and matching pairs on 1:1, **Supplementary Table 1** outlines the demographic and clinical characteristics of the paired sample (2213 patients in each group). Compared with the comparison results of the previously unmatched group, there were differences in gender, age, marital status, use of antihypertensive drugs, use of lipid-lowering drugs, and ADL total at discharge among the paired samples, while other results were generally consistent.

**Figure 1:**
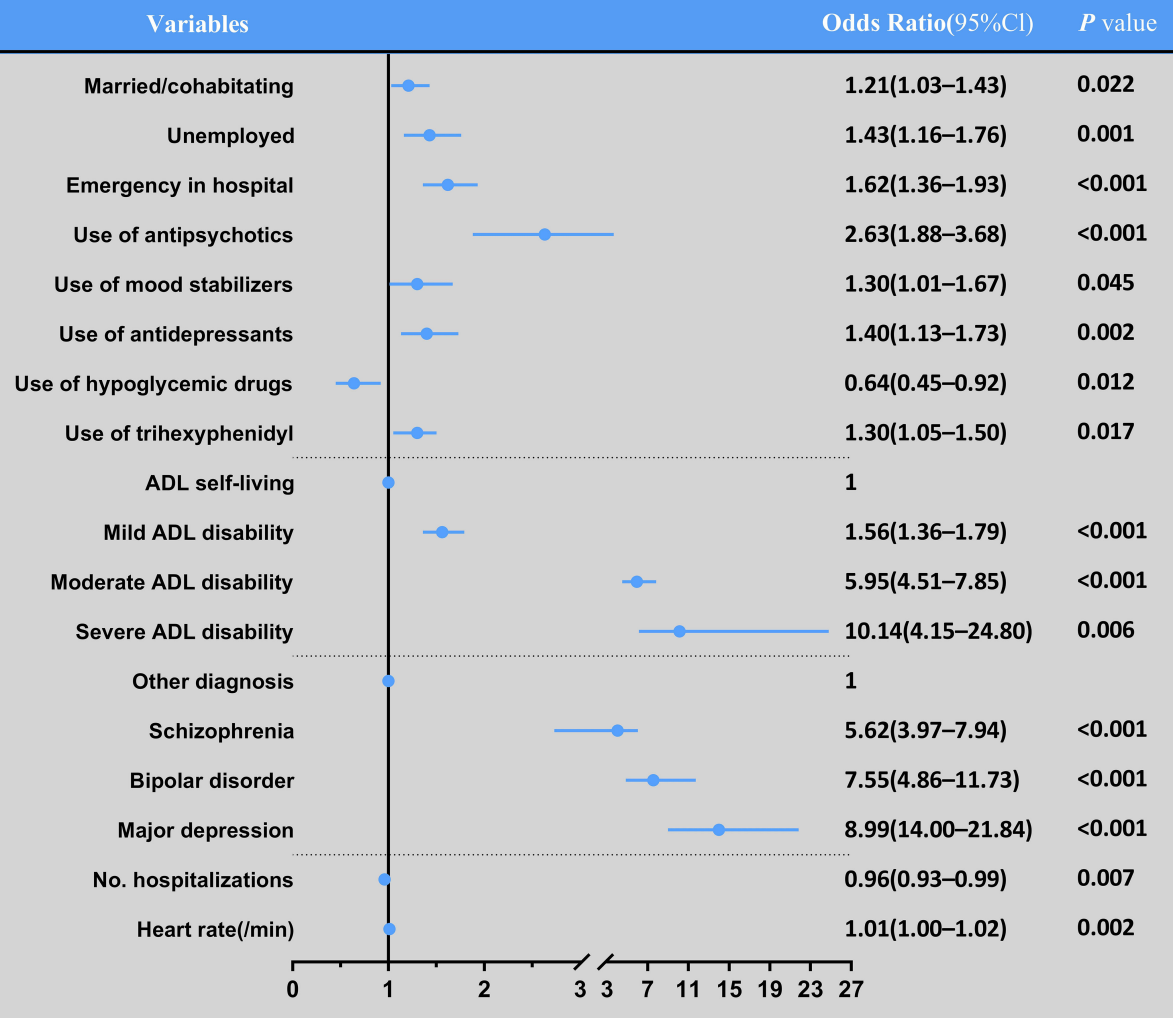
Independent contributors to ECT (multiple logistic regression analysis).

Conditional logistic regression analysis showed that married/cohabitating, unemployed, emergency admissions were significantly associated with the use of ECT (OR_1_ = 1.21, 95% CI_1_: 1.03-1.43) (OR_2_ = 1.43, 95% CI_2_: 1.16-1.76) (OR_3_ = 1.62, 95% CI_3_: 1.36-1.93). The use of antipsychotics, mood stablilizers, antidepressants and trihexyphenidyl were also significantly associated with the use of ECT (OR_1_ = 2.63, 95% CI_1_: 1.88-3.68) (OR_2_ = 1.30, 95% CI_2_: 1.01-1.67) (OR_3_ = 1.40, 95% CI_3_: 1.13-1.73) (OR_4_ = 1.30, 95% CI_4_: 1.05-1.50). Patients with mild, moderate and severe ADL disability had 1.56 times (95% CI: 1.36-1.79), 5.95 times (95% CI: 4.51-7.85), 10.14 times (95% CI: 4.15-24.80) higher risk of using ECT compared to the ADL self-living individuals. Patients with diagnosing schizophrenia, bipolar disorder and major depression had 5.62 times (95% CI: 3.97-7.94), 7.55 times (95% CI: 4.86-11.73), 8.99 times (95% CI: 14.00-21.84) higher risk of using ECT compared to the individuals with other diagnosis. The use of hypoglycemic drugs (OR = 0.64, 95% CI: 0.45-0.83), hospitalizations (OR = 0.96, 95% CI: 0.93-0.99) were negatively associated with the use of ECT. The effect of heart rate was relatively small but still statistically significant (OR = 1.01, 95% CI: 1.01-1.02) (**Figure 1**).

**Figure 2:**
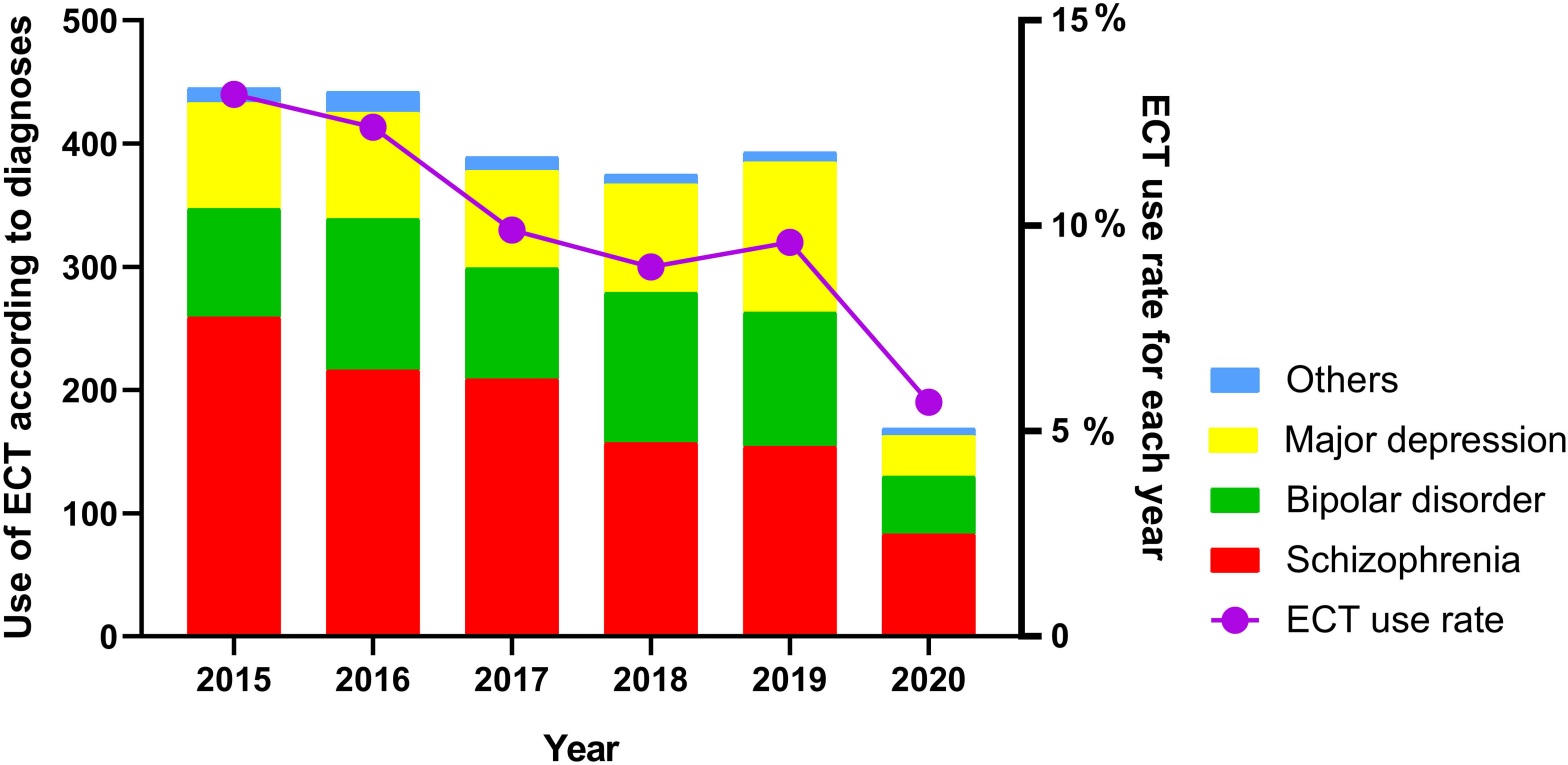
Use of ECT according to diagnoses and ECT use rate by year.

In addition, compared to ECT use on 2015, ECT use decreased significantly from 2016 to 2020. In 2020, when all aspects of life were negatively affected by the COVID-19 pandemic in China, the number of patients receiving ECT treatment was the lowest (**Figure 2**).

## Discussion

This observational study found that 10.0% of 22,120 inpatients in Beijing’s largest Grade A tertiary psychiatric hospital between 2016 and 2020 received ECT. We analyzed the largest single center ECT data in China in the past eight years. In contrast to previous studies, our research included detailed basic demographic and clinical characteristics as well as analyzed the vital signs and ADL ability of inpatients. Finally, we found that the receipt of ECT was independently associated with Married/cohabitating; few hospitalizations; unemployed; emergency referral; reduced use of hypoglycemic drugs; fast heart rate; severe ADL disability; and primary diagnoses of BD, MDD, and SCH.

We found that 10% of the inpatients received ECT, and this rate is slightly lower than that reported at a Chinese psychiatric hospital outside the Beijing area (17.8%) (12). Moreover, another study conducted in the Beijing area reported an ECT rate of 57.7% (11), and the frequency of ECT use in our study was closer to that reported in foreign countries: 1.2–7.4% in the USA, 13.6% in Japan, 13.8% in India, and 6% in Denmark (10, 20–25). This difference in the rates between China and these countries can be attributed to an inconsistent understanding of ECT indications among different hospitals. In addition, our treatment principle of focusing on early prevention of mental illness and reducing the possibility of severe mental diseases requiring ECT may also explain some of these differences. Of note, the annual decline in ECT use in our study was roughly consistent with the global trends (10, 26). In particular, the lowest uptake of ECT was reported in 2020 – when the COVID-19 pandemic impacted ECT services worldwide. During the COVID-19 period, several realistic factors limited the use of ECT, such as: the ECT operation room needing longer time for disinfection (about twice the usual time), patients without the cold symptoms (not considered as a source of viral infection), manpower shortage (many doctors were appointed to service nucleic acid testing), and patients restricted in their own wards (to reduce virus transmission) (27, 28).

ECT is commonly assumed to be a primary (first-line) treatment with strict indications, referring to the clinical guidelines of five authoritative psychiatric associations (American Psychiatric Association, Canadian Network for Mood and Anxiety Treatments, The Royal Australian and New Zealand College of Psychiatrists, The Royal College of Psychiatrists, and World Federation of Societies of Biological Psychiatry) (29–33). In China, patients with high suicide/aggressive behavior risk, food/fluid refusal, stupor, and extreme lack of cooperation or treatment resistance are given priority for ECT (34). Similar to our findings, ECT is used more frequently for BD or MDD inpatients (35). According to authoritative reviews and international guidelines, ECT can also be used to relieve symptoms of treatment-resistant schizophrenia; however, attention should be paid to its benefits and side effects.

From the initial results, we speculate that younger and female patients were more likely to receive ECT, which is consistent with other results (12), Demographically, there was a predominance of female patients of 53.4% in our results, 51% in Japan (36), 56% in Pakistan (37), 60% in Saudi Arabia (38), 71% in Australia (39), 57.7%-60.1% in Sweden (40, 41), 61.0% in Poland (42) and 57% in Norway (43). Our analysis of sex distribution in affective disorders showed that there were a higher proportion of female, up to 62%. We realize that the higher proportion of female in ECT is a global trend, especially in Western countries where ECT originated (44). This may be related to factors such as the higher prevalence of mental disorders among women, their susceptibility to hormones and environmental variability (45). On the contrary, in several African regions (29%-46%) (46, 47) and other Asian countries [28% in Katmandu (48); 39% in India (49)], the proportion of women in ECT is relatively low. This the specific reasons maybe need further investigations. However, from another perspective, the above different results are not inconsistent and may be related to bias and confounding factors. Therefore, we further conducted PSM analysis based on gender and age, with matching pairs on 1:1.

After PSM analysis, the figure clearly shows that the use of ECT was independently associated with fewer hospitalizations, confirming earlier results that ECT may reduce readmission rates through satisfactory treatment outcomes (50). Our analysis showed that the status of being married was associated with the use of ECT. One possible explanation is that being married was associated with family members recognizing of faster disease relief through the use of ECT (51). We speculate that unemployment may be an aspect of impaired social functioning in patients with mental illness. This suggested that the disease may be more severe and patients may be more likely to choose ECT to get quickly improvement and restore their health (52).

Notably, this is the first retrospective study to explore the relationship between the use of ECT and severity (including assessment of emergency, vital signs, and physical prescriptions). The results showed that more than one-third of the patients in the ECT group were referred from emergency departments. Emergency hospitalization may provide a fast and effective therapeutic opportunity for patients at risk, including receiving ECT services. In previous studies, the heart rate was associated with disease severity and urgency (53, 54). In addition, the different result of hypoglycemic drugs in **Figure 1** maybe need further research to verify the differences (55).

Diminished ADL is often regarded as a characteristic feature of severe mental disorders (56), such as the incapacity of individuals with profound depression or impulsivity to effectively accomplish certain activities in their daily lives (57). Consistent with our viewpoint, another study found the patients with ECT had worse ADL score compared to those without ECT. As shown in our results, the degree of ADL disability at admission is a positive factor for predicting acceptance of ECT treatment (58). Previous studies have shown that psychiatric patients in the acute phase have varying degrees of impairment in the self-care and daily life (59). During the rehabilitation period, except for severe organic psychiatric disorders, the impaired daily living abilities will get recovery for most patients (60). This is as observed in **Table 1** of this article.

### Limitations

This study had several limitations. First, the results of this retrospective study should be considered with caution owing to methodological limitations. Second, the data were collected from a single center, and the findings are not entirely applicable to other regions of China. Third, although our study included relevant variables such as non-psychiatric prescriptions and vital signs, evaluation data on the severity of psychiatric symptoms were still lacking. Finally, some specific indications like catatonia were not been extracted, and might be classified as special symptoms of a disease, not been independently diagnosed. Nevertheless, we analyzed a large dataset covering 6 consecutive years to determine the pattern of ECT use and the factors correlated with ECT usage from a large psychiatric center in China over the last years.

## Conclusion

In conclusion, this study found that the usage rate and yearly decreasing trend in ECT use in the largest tertiary psychiatric center in Beijing were roughly consistent with the recent trends in other regions. ECT use was associated with married/cohabitating; unemployed; few hospitalizations; emergency referral; increased use of antipsychotics, mood stabilizers, antidepressants; reduced use of hypoglycemic drugs; fast heart rate; and severe ADL disability. Given the variations in previous research findings, it is crucial for clinical psychiatrists to adopt a scientific approach and the evidence based practice when establishing ECT protocols that adhere to the ECT utilization standards.

## Data Availability

The raw data supporting the conclusions of this article will be made available by the authors, without undue reservation.

## Supplementary material

Supplementary Table 1 Demographic and characteristics of the two groups by propensity score matching (PSM).
