# Supplementary material for: Trend of electroconvulsive therapy use and its relationships with clinical characteristics from a large psychiatric center in China
Source: Front Psychiatry. 2025 Jun 3;16:1508044. doi: 10.3389/fpsyt.2025.1508044 (PMC12170517; doi:10.3389/fpsyt.2025.1508044)
Supplement: Supplementary Table 1 — Demographic and characteristics of the two groups by propensity score matching (PSM). [file Table1.doc]

**Supplement Table1** Demographic and characteristics of the two groups by propensity score matching (PSM) .

|  | **Non-ECT group**  **（n=2213）** | | **ECT group**  **（n=2213）** | | **Statistics** | | |
| --- | --- | --- | --- | --- | --- | --- | --- |
|  | **N** | **%** | **N** | **%** | ***X^2^*** | **Df** | ***P*** |
| Male, N(%) | 832 | 37.6 | 811 | 36.6 | 0.4 | 1 | 0.51 |
| Education（Undergraduate） | 1062 | 48.0 | 1099 | 49.7 | 0.7 | 1 | 0.40 |
| Married/cohabitating | 952 | 46.4 | 1110 | 50.2 | 6.2 | 1 | 0.01 |
| Han nationality | 2161 | 97.7 | 2165 | 97.8 | 0.2 | 1 | 0.69 |
| Religion（No religious belief） | 2086 | 94.3 | 2080 | 94.0 | 0.1 | 1 | 0.75 |
| Local resident | 1103 | 49.8 | 946 | 42.7 | 22.4 | 1 | <0.001 |
| Employed | 1907 | 86.2 | 1765 | 79.8 | 32.2 | 1 | <0.001 |
| Emergency in hospital | 521 | 23.5 | 802 | 36.2 | 85.1 | 1 | <0.001 |
| Primary psychiatric diagnosis |  |  |  |  | 342.3 | 1 | <0.001 |
| Schizophrenia-spectrum disorders | 1366 | 61.7 | 1078 | 48.7 |  |  |  |
| Bipolar disorder | 345 | 15.6 | 579 | 26.2 |  |  |  |
| Major depression | 218 | 9.9 | 494 | 22.3 |  |  |  |
| Others | 284 | 12.8 | 62 | 2.8 |  |  |  |
| Psychiatric drugs |  |  |  |  |  |  |  |
| Use of antipsychotics | 1976 | 89.3 | 2060 | 93.1 | 19.8 | 1 | <0.001 |
| Use of mood stabilizers | 387 | 17.5 | 692 | 31.3 | 114.1 | 1 | <0.001 |
| Use of antidepressants | 603 | 27.2 | 860 | 38.9 | 67.4 | 1 | <0.001 |
| Use of cognitive-enhancing drug | 9 | 0.4 | 2 | 0.1 | 4.5 | 1 | 0.04 |
| Use of trihexyphenidyl | 744 | 33.6 | 858 | 38.8 | 12.7 | 1 | <0.001 |
| Physical disease drugs |  |  |  |  |  |  |  |
| Use of antihypertensive drugs | 137 | 6.2 | 159 | 7.2 | 1.8 | 1 | 0.19 |
| Use of lipid-lowering drugs | 388 | 17.5 | 425 | 19.2 | 2.1 | 1 | 0.15 |
| Use of hypoglycemic drugs | 182 | 8.2 | 94 | 4.2 | 29.9 | 1 | <0.001 |
|  | **M** | **SD** | **M** | **SD** | ***Z/t*** |  | ***P*** |
| Age | 36.1 | 12.2 | 35.7 | 12.2 | 1.3 |  | 0.21 |
| Current hospital stay (days) | 68.9 | 60.7 | 66.5 | 59.7 | 1.4 |  | 0.17 |
| No. of hospitalizations | 3.3 | 3.2 | 2.6 | 3.3 | 6.9 |  | <0.001 |
| Heart rate (/min) | 81.6 | 10.2 | 83.0 | 12.1 | -3.4 |  | 0.001 |
| SBP before ECT course (mmHg) | 116.9 | 12.2 | 117.4 | 13.9 | -1.3 |  | 0.20 |
| DBP before ECT course (mmHg) | 76.0 | 8.3 | 76.0 | 9.4 | -0.2 |  | 0.89 |
| ADL total at admission | 91.7 | 8.7 | 87.2 | 13.1 | 13.52 |  | <0.001 |
| ADL total at discharge | 97.0 | 6.6 | 96.9 | 7.6 | 0.5 |  | 0.63 |
| Changes in ADL total | 5.3 | 7.3 | 9.7 | 12.3 | -14.5 |  | <0.001 |
| No. Sessions of ECT |  |  | 10.3 | 6.6 |  |  |  |
| The intensity of ECT (J) |  |  | 27.7 | 15.2 |  |  |  |
| Seizure duration (s) |  |  | 49.2 | 23.1 |  |  |  |

SBP: systolic blood pressure; DBP: diastolic blood pressure; ADL: daily living abilities;
